# Sign-reversed anomalous Nernst effect with matched Seebeck coefficient in lanthanide-iron alloys for the direct sensing of heat flux

**DOI:** 10.1080/14686996.2025.2544649

**Published:** 2025-08-07

**Authors:** Hyun Yu, Sang J. Park, Inho Lee, Ji Hoon Shim, Hyungyu Jin

**Affiliations:** a Department of Mechanical Engineering, Pohang University of Science and Technology (POSTECH), Pohang, South Korea; b Department of Chemistry, Pohang University of Science and Technology (POSTECH), Pohang, South Korea

**Keywords:** Heat flux sensor, anomalous Nernst effect, anomalous Nernst thermopile, lanthanide-iron alloy, uncertainty of heat flux sensing

## Abstract

Heat flux sensors based on the anomalous Nernst effect (ANE) have emerged as a promising solution for achieving thin and flexible designs. ANE-based heat flux sensors typically employ thermopile structures composed of two ANE materials with opposite signs, connected in series to enhance sensing performance. However, a mismatch in the Seebeck coefficient between the two ANE materials causes a considerable offset voltage due to the Seebeck effect (SE) under oblique heat flux. This parasitic sensing voltage hinders direct sensing of heat flux in the intended direction. In this study, a sign-reversed ANE with matched Seebeck coefficient is examined in Fe_3_Ln (Ln = Gd, Tb, Dy, Ho, and Er), enabling a thermopile structure free from the SE-induced offset voltage. Based on density functional theory calculations, Fe₃Ln is selected as a suitable candidate for exhibiting sign reversal of ANE while maintaining the Seebeck coefficient. At 300 K, Fe_3_Ln (Ln = Gd, Tb, Dy, and Ho) exhibits a positive ANE sign, whereas Fe_3_Er exhibits a negative ANE sign, facilitating the combination of two sign-reversed ANE materials. Among these, Fe_3_Ho and Fe_3_Er demonstrate the lowest Seebeck coefficient difference of 0.45 μV K^−1^, minimizing the offset voltage-induced relative uncertainty, as confirmed by COMSOL simulations – comparable to that of other SE-based heat flux sensors. This study paves the way for the development of ANE-based heat flux sensors by introducing a novel approach to pairing opposite-ANE-sign materials with matched Seebeck coefficient, enabling direct and accurate heat flux sensing via thermopile structures.

## IMPACT STATEMENT

This study presents a sign-reversed anomalous Nernst effect with matched Seebeck coefficients in Fe_3_Ln, enabling direct and accurate heat flux sensing while minimizing offset voltage-induced relative uncertainty.

## GRAPHICAL ABSTRACT

**Figure:**
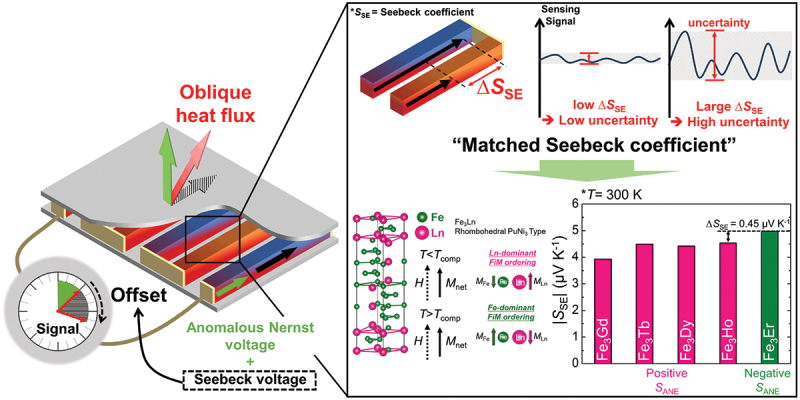

## 1. Introduction

Heat flux sensors (HFSs) have attracted significant interest due to their potential applications in thermal management, such as battery systems and healthcare devices [1–3]. HFSs enable accurate analysis of thermal properties, including heat absorption, dissipation, and internal temperature measurements, which are often challenging to achieve with conventional temperature sensors [4]. Traditionally, HFSs have been developed based on the Seebeck effect (SE), where a longitudinal thermoelectric voltage is generated in response to a heat flux. However, SE-based HFSs require multiple pairs of π-shaped thermoelectric legs connected in series, resulting in complex device structures. Additionally, the heat flux sensitivity of SE-based HFS (*G*_SE_) is proportional to the thickness of the thermoelectric leg ($L_{⊥}$) [5]. As a result, SE-based HFSs face challenges when integrated into embedded systems within advanced electronics and flexible devices. Recently, anomalous Nernst effect (ANE)-based HFSs have emerged as an alternative, offering simplified device geometries compared to SE-based HFSs [6–10]. The ANE generates a transverse thermoelectric voltage perpendicular to the temperature gradient and an externally applied magnetic field. Because of its voltage generation geometry, the heat flux sensitivity of ANE-based HFS (*G*_ANE_) is proportional to the transverse length of the thermoelectric leg ($L_{∥}$). Accordingly, *G*_ANE_ can be increased by enlarging $L_{∥}$ without requiring a complex structure (e.g. numerous π-shaped pairs in the SE-based HFS). Moreover, since *G*_ANE_ is independent of $L_{⊥}$, ANE-based HFSs can achieve high sensitivity even in thin-film configurations. These advantages enable ANE-based HFSs to be designed with simplicity and flexibility, making them well-suited for efficient heat management across diverse applications.

ANE-based HFSs have been demonstrated in previous studies using an anomalous Nernst thermopile (ANT), which consists of two ANE materials with opposite signs connected in series to enhance *G*_ANE_ [11–14]. While this design permits cumulative increases in *G*_ANE_ by enhancing the effective transverse voltage output, significant uncertainty in the target direction caused by the SE-based offset voltage (*V*_offset_) may arise under oblique heat flux if the constituent materials exhibit considerable differences in their Seebeck coefficients (∆*S*_SE_) [12]. The offset voltage-induced relative uncertainty (*u*_r_) under oblique heat flux is calculated as the ratio of the *V*_offset_ and the ANE-based signal voltage (*V*_signal_), expressed as:

(1) $$u_{r}=\frac{V_{\mathrm{offset}}}{V_{\mathrm{signal}}}=\frac{\Delta V_{\mathrm{SE}}}{\Delta V_{\mathrm{ANE}}}=\frac{\Delta S_{\mathrm{SE}}}{\Delta S_{\mathrm{ANE}}}\times \frac{L_{⊥}}{L_{∥}}\times \frac{\Delta T_{∥}}{\Delta T_{⊥}}$$

where ∆*S*_ANE_ represents the differences in anomalous Nernst coefficients between two ANE materials, while $T_{∥}$ and $T_{⊥}$ denote the lateral and perpendicular temperature differences, respectively. (a full derivation is given in Supplemental material Note 1 and Figure S1) [15]. Equation (1) indicates that *u*_r_ strongly depends not only on ∆*S*_ANE_ but also on ∆*S*_SE_. Since *S*_SE_ is typically 1 to 2 orders of magnitude higher than *S*_ANE_ in ANE materials [16–25], the mismatch of *S*_SE_ can cause a considerable *V*_offset_ to contaminate *V*_signal_ and further increases *u*_r_ under even a small amount of oblique heat flux. For example, Bang et al. reported a large *V*_SE_ in the transverse direction comparable to *V*_ANE_ under oblique heat flux in a titled structure [26]. Therefore, matching the *S*_SE_ of the two sign-reversed ANE materials is essential for reducing *u*_r_ and achieving direct and accurate heat flux sensing using the ANT – a key factor that has been largely overlooked so far.

Compensated ferrimagnetic lanthanide (Ln)-transition metal (TM) alloys have been proposed as materials capable of reversing the sign of *S*_ANE_ while exhibiting a small ∆*S*_SE_ [13,14,27,28]. These alloys enable the reversal of the sign of *S*_ANE_ within the same alloy system, eliminating the need to combine different ANE materials with opposite signs (e.g. FePt/MnGa) [11] and more effectively reducing ∆*S*_SE_. In conventional ANE materials [16–25], the sign of *S*_ANE_ typically depends on the direction of net magnetization. In Ln-TM alloys, *S*_ANE_ is primarily governed by the magnetic moments of TM atoms, as their 3d orbitals near the Fermi level (*E*_F_) strongly influence transport properties, while the 4f orbitals of Ln atoms, far from the *E*_F_, contribute negligibly. As the relative magnitude of the magnetic moment between Ln and TM atoms varies, the direction of the magnetic moments of TM atoms can reverse even if the net magnetization remains unchanged, leading to a corresponding sign change in *S*_ANE_. For example, Odagiri et al. observed sign reversal of *S*_ANE_ with a small ∆*S*_SE_ in GdCo and TbCo alloys by adjusting the Ln/TM composition [14], achieving a ∆*S*_ANE_ of 2 μV K^−1^ and a ∆*S*_SE_ of 2 μV K^−1^ in Gd_15.5_Co_84.5_/Gd_23.7_Co_76.3_. However, modifying the TM composition – which significantly affects transport properties – inevitably introduces fluctuations in *S*_SE_, highlighting the need for further refinement. In contrast, sign reversal through the substitution of the Ln element, which has a negligible effect on transport properties, is expected to address this issue, thereby minimizing *u*_r_ under oblique heat flux.

In this study, we investigate compensated ferrimagnet Fe_3_Ln (Ln = Gd, Tb, Dy, Ho, and Er) as a potential sign-reversed ANE material with matched *S*_SE_. X-ray diffraction (XRD) and magnetic measurements confirmed that the Fe_3_Ln maintains its crystal structure while exhibiting a tunable compensation temperature (*T*_comp_), leading to a sign reversal of *S*_ANE_ by substituting the Ln site. Density functional theory (DFT) calculations of density of states (DOS) and band structure further confirmed that the sign reversal of *S*_ANE_ occurs without significant changes in *S*_SE_. Indeed, the replacement of Ln resulted in a sign reversal of *S*_ANE_ while maintaining a comparable magnitude of *S*_SE_ at 300 K. Specifically, Fe_3_Ln (Ln = Gd, Tb, Dy, and Ho) and Fe_3_Er exhibited positive and negative signs of *S*_ANE,_ respectively, with a notably small ∆*S*_SE_ of 0.45 μV K^−1^ between Fe_3_Ho and Fe_3_Er. The combination of Fe_3_Ho and Fe_3_Er exhibited a ∆*S*_SE_/∆*S*_ANE_ of 0.64, which is significantly smaller than other combinations of reversed sign ANE materials [11,13,29]. Subsequently, COMSOL simulations were conducted to analyze *u*_r_ under oblique heat flux conditions. The ANT composed of Fe_3_Ho and Fe_3_Er achieved an *u*_r_ of less than 5.5% until $Q_{∥}$ reaches the same value as $Q_{⊥}$, a performance comparable to that of other SE-based HFSs [30–32].

## 2. Experimental procedures

### 2.1. Fabrication and characterization

Polycrystalline Fe_3_Ln (Ln = Gd, Tb, Dy, Ho, and Er) was fabricated by arc melting a mixture of high-purity raw materials (Gd (4N), Tb (4N), Dy (4N), Ho (4N), and Er (4N), Merck) under an Ar atmosphere. Arc-melted alloys were sealed in a quartz tube under a high vacuum ( <10^−5^ Torr) and annealed for a week at 1073 K to ensure homogeneity. Next, the quartz tube was quenched with cold water, and the polycrystalline alloys were cut into several pieces using a diamond wire saw for subsequent characterization and transport measurements. XRD measurements (D/MAX-2500-PC, Rigaku, Japan) with Cu K_α_ radiation at 40 kV and 40 mA from 2θ = 20° to 80° revealed a rhombohedral PuNi_3_-type crystal structure.

### 2.2. Magnetic properties measurement

A vibrating sample magnetometer (MPMS3-Evercool, Quantum Design Inc., USA) was utilized to perform magnetic measurements. The temperature dependence of the magnetization was measured at a magnetic field of 2 T in the temperature range from 10 to 400 K, and the magnetic field dependence of the magnetization was measured in a magnetic field from − 2 to 2 T at 80 K, 300 K, and the *T*_comp_.

### 2.3. Density function theory calculation

DFT calculations were performed using the full-potential linearized augmented plane-wave method (PAW) implemented in the WIEN2k package [33]. The exchange-correlation functional of the generalized gradient approximation (GGA) with Perdew – Burke – Ernzerhof parameterization (PBE) was adopted [34]. The DFT calculations included both the Coulomb correlation and spin – orbit interactions within the GGA+U+SO approximation. In this case, 1000 k-points in the first Brillouin zone were used, with U = 7 eV for Ln-4f states. A U value of approximately 7 eV is commonly adopted in literature, as it has been shown to reasonably reproduce the experimental electronic and magnetic properties of rare-earth-based systems [35,36].

### 2.4. Transport properties measurement

A customized liquid-nitrogen cryostat system (Janis Cryostat, Lake Shore Cryotronics Inc., USA) was used to measure electric and thermal transport properties. Two current inputs were attached to the top and bottom of the rectangular bar-shaped samples (2.5 mm × 3.5 mm × 5.5 mm). Four voltage leads were attached to the two edges on opposite sides of the samples. The standard four-probe method was used to measure electrical and Hall resistances. The T-type thermocouples were attached on the two edges of one side to estimate the temperature difference. Two 120-Ω resistive heaters connected in series were attached above the samples. Under a heat flux applied by the heaters into the samples, SE voltage and Nernst voltage were measured, whereas magnetic field *H* was continuously swept between ±1.25 T at approximately 7 mT s^−1^. All measurements were performed under a high vacuum ( <10^−5^ Torr), and the measurement setups were covered with a gold-plated radiation shield to minimize convective and radiative heat losses. Additional experimental details have been presented in previous studies [37,38].

### 2.5. Numerical simulation

COMSOL Multiphysics (version 6.2) was used to simulate the *u*_r_ of the ANT. A simplified geometry and boundary conditions are described in Supplemental Note 2 and Figure S2. The uncertainties for various $Q_{∥}$/$Q_{⊥}$ and ∆*S*_SE_/∆*S*_ANE_ were then calculated based on the material properties of Fe_3_Ho and Fe_3_Er.

## 3. Results

### 3.1. Candidate material for direct heat flux sensing via the anomalous Nernst thermopile

**Figure 1.**
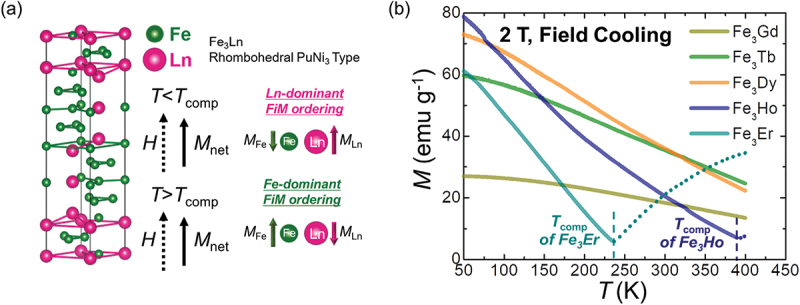
(a) Crystal structure of the rhombohedral PuNi_3_-type phase and ferrimagnetic ordering below and above *T*_comp_. Green and pink spheres represent Fe and Ln atoms, respectively. *M*_Fe_, *M*_Ln_, and *M*_net_ denote the magnetic moments of the Fe atoms, Ln atoms, and the net magnetic moment, respectively. (b) Temperature dependence of the field-cooled magnetization and *T*_comp_ (represented by the dashed line).

We selected compensated ferrimagnetic Fe_3_Ln (Ln = Gd, Tb, Dy, Ho, and Er) as a candidate material for sign-reversed ANE with matched *S*_SE_. Fe_3_Ln has been reported to exhibit reversed FiM orderings, Ln-dominant below and Fe-dominant above *T*_comp_ [39], as illustrated in Figure 1(a). This behavior is consistent with other compensated ferrimagnetic Ln-TM alloys [13,14,27,28,40,41]. Similar to these alloys, Fe_3_Ln is expected to demonstrate a sign reversal of *S*_ANE_ due to the flipping of the magnetic moment of the TM atoms, particularly Fe in this system. Specifically, the Ln-dominant and Fe-dominant FiM orderings, which exhibit opposite directions of Fe magnetic moments, result in *S*_ANE_ with opposite signs. Furthermore, the Ln site can be substituted with various elements (e.g. Gd, Tb, Dy, Ho, and Er), allowing *T*_comp_ to be tailored while maintaining the PuNi_3_-type crystal structure. Engineering *T*_comp_ by replacing the Ln site enables control over FiM ordering at specific temperatures – whether Ln-dominant or Fe-dominant – thereby facilitating the reversal of *S*_ANE_ [39].

**Figure 2.**
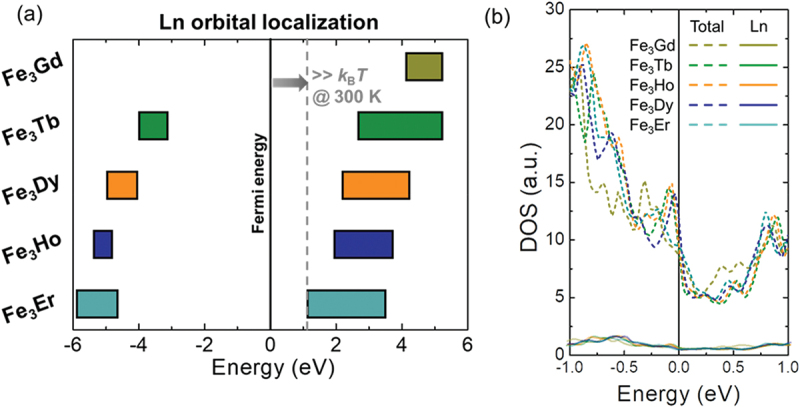
(a) Localized orbitals of Ln. Orbital localization of Fe_3_Ln is shown as bars as a function of energy for visual comparison. *k*_B_T at 300 K is indicated by the gray dashed line. (b) Total- and Ln- DOS near the *E*_F_.

We confirmed that *T*_comp_ is engineered by substituting the Ln site while maintaining the crystal structure through XRD and magnetic property measurements. The XRD patterns in Figure S5 show that all Fe_3_Ln compounds retain a PuNi_3_-type crystal structure with only negligible secondary peaks near 39° and 42°, corresponding to a minor Th_6_Mn_23_-type phase. *M-T* curves in Figure 2(b) indicate that Fe_3_Ho and Fe_3_Er exhibit magnetic compensation at *T*_comp_ of 236.3 K and 392 K, respectively. In contrast, Fe_3_Gd, Fe_3_Tb, and Fe_3_Dy do not exhibit magnetic compensation within the measurable temperature range (up to 420 K) because of their high *T*_comp_, which is consistent with a previous study [39]. Accordingly, Fe_3_Er (with *T*_comp_ below 300 K) is expected to exhibit Fe-dominant FiM ordering, whereas the other Fe_3_Ln compounds (with *T*_comp_ above 300 K) exhibit Ln-dominant FiM ordering, leading to the reversal of *S*_ANE_ between Fe_3_Er and the other compounds at 300 K.

Subsequently, the preservation of *S*_SE_ in Fe_3_Ln was evaluated through an analysis of the band structure and DOS obtained from DFT calculations. The band structures and the Spin-DOS of Fe_3_Ln illustrate the Ln and Fe orbitals, represented by red and blue lines, respectively, in Figures S6 and S7. Figure 2(a) shows the localized Ln orbitals in all Fe_3_Ln, which are far away from the *E*_F_, significantly larger than the *k*_B_*T* of 0.025 eV at room temperature (*k*_B_: Boltzmann constant). To further examine the contribution of Ln orbital near *E*_F_ that directly influences transport, Figure  2(b) presents the total and Ln-DOS. While the total DOS exhibits non-negligible fluctuations, primarily originating from Fe orbital, these variations can inevitably arise from indirect effects of Ln substitution, such as shifts in chemical potential or changes in lattice parameters. In contrast, the Ln-DOS remains consistently small in magnitude and exhibits minimal variation across different Ln elements. This observation suggests that the direct contribution of Ln orbital to the *S*_SE_ is limited, reinforcing the conclusion that *S*_SE_ is preserved upon Ln substitution in Fe_3_Ln.

### 3.2. Sign reversed anomalous Nernst effect with matched Seebeck coefficient

**Figure 3.**
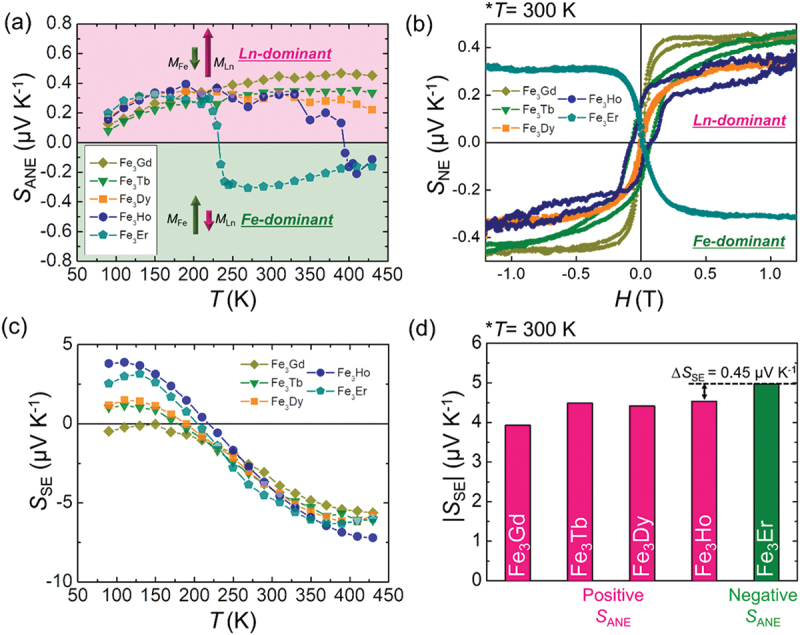
(a) Temperature dependence of *S*_ANE_. *S*_ANE_ in the pink regime indicates ln-dominant ordering, whereas *S*_ANE_ in the green regime indicates Fe-dominant ordering. (b) Magnetic field dependence of the Nernst coefficient *S*_NE_ at 300 K. (c) Temperature dependence of *S*_SE_. (d) Comparison of *S*_SE_ at 300 K. Pink bars denote a positive sign of *S*_ANE_, while green bar denotes a negative sign of *S*_ANE_.

Next, we investigated the transport properties of Fe_3_Ln. Figure 3(a) shows the temperature dependence of *S*_ANE_ measured from 80 to 420 K. Indeed, a clear sign reversal is observed above *T*_comp_ in Fe_3_Ho and Fe_3_Er, where the FiM ordering transitions from Fe-dominant to Ln-dominant. Specifically, *S*_ANE_ is positive for Ln-dominant FiM ordering and negative for Fe-dominant FiM ordering. By substituting Ln, the sign of *S*_ANE_ can be reversed by modifying the FiM ordering at specific temperatures. At 300 K, Fe_3_Er, which exhibits Fe-dominant FiM ordering, exclusively shows a negative *S*_ANE_, whereas the other Fe_3_Ln compounds with Ln-dominant FiM ordering display a positive *S*_ANE_, as shown in Figure 3(b). Despite the absence of a change in the direction of net magnetization, as depicted in the *M – H* curves (Figures 2(b) and S8), the sign of *S*_ANE_ varies depending on the magnetic moment of TM atoms through Ln substitution. The sign reversal of *S*_ANE_ is further examined using the anomalous Hall effect (AHE), the electrical counterpart of ANE. Figure S9(a) shows that the anomalous Hall coefficient (*ρ*_AHE_) reverses its sign above *T*_comp_ in Fe_3_Ho and Fe_3_Er, consistent with *S*_ANE_. Additionally, at 300 K, Fe_3_Er and Fe_3_Ho also exhibit a reversed sign of *ρ*_AHE_, as shown in Figure S9(b). This supports the conclusion that the sign reversal of *S*_ANE_ is driven by the magnetic moment of TM atoms rather than the net magnetization. As predicted by DFT calculations, the substitution of Ln in Fe_3_Ln does not significantly affect *S*_SE_. Figure 3(c) illustrates the temperature dependence of *S*_SE_ measured from 80 to 420 K for Fe_3_Ln. All Fe_3_Ln compounds exhibit a similar *S*_SE_ trend, maintaining a comparable magnitude within the range of 4 to −7 μV K^−1^. Below 200 K, *S*_SE_ fluctuations become more pronounced, occasionally exhibiting sign reversals. This behavior can be attributed to additional scattering mechanisms, including phonon and magnon drag effects, observed in many magnetic metals [42,43]. Notably, Fe_3_Ho and Fe_3_Er, which exhibit reversed signs of *S*_ANE_ and similar *S*_SE_ over a wide temperature range (250–380 K), show only a minimal ∆*S*_SE_ of 0.45 μV K^−1^ at 300 K, as shown in Figure 3(d) and Table S1. Furthermore, selecting Ln elements with different *T*_comp_, such as Fe_3_Gd, which exhibits a higher *T*_comp_ (~600 K) than Fe_3_Ho, allows expansion of the effective operating temperature range. Despite its inherent advantages, the observed *S*_ANE_ values remain relatively modest (0.29–0.45 μV K^− 1^) compared to other ANE materials (Table S1) [16–25], with the maximum Δ*S*_ANE_ among the Fe_3_Ln reaching only ~0.73 μV K^− 1^. This limitation may constrain HFS performance in certain applications. However, the band structure of Fe_3_Ln suggests the presence of topological features, such as Weyl points near the *E*_F_ (Figure S10), that are associated with high Berry curvature and transverse thermoelectric performance [18,19]. This indicates strong potential for enhancement via band engineering. Approaches such as chemical substitution, strain tuning, or spin-orbit coupling modulation may further increase *S*_ANE_ without compromising *S*_SE_ stability, thereby enabling improved ANT-based heat flux sensing performance.

### 3.3. Numerical simulation of the offset voltage-induced relative uncertainty

**Figure 4.**
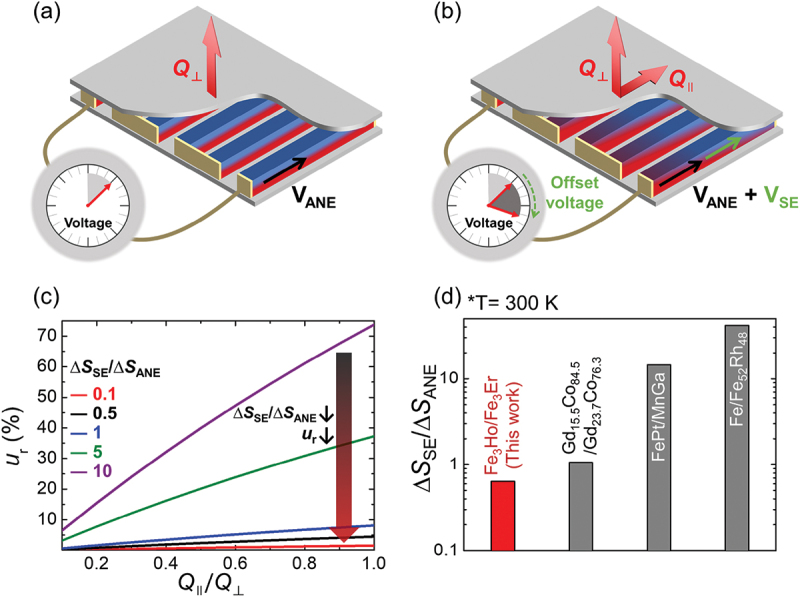
Schematic of SE-based offset voltage generation under (a) $Q_{⊥}$ and (b) both $Q_{∥}$ and $Q_{⊥}$. (c) Numerical simulation of *u*_r_ with change in ∆*S*_SE_/∆*S*_ANE_, (d) ∆*S*_SE_/∆*S*_ANE_ of an ANT composed of Fe_3_Ho and Fe_3_Er and previously reported ANE materials of opposite sign at 300 K.

The *u*_r_ of the ANT was analyzed using COMSOL simulations (detailed in the experimental section). Figure 4(a,b) depict schematics of transverse voltage generation under various heat conditions: (a) $Q_{⊥}$ and (b) both $Q_{∥}$ and $Q_{⊥}$. While *V*_ANE_ is read out as *V*_signal_ under $Q_{⊥}$ (Figure 4(a)), both *V*_signal_ and *V*_offset_ are obtained from the sum of *V*_ANE_ from $Q_{⊥}$ and *V*_SE_ from $Q_{∥}$ under both $Q_{∥}$ and $Q_{⊥}$ (Figure 4(b)). Figure S11(a) depicts the numerical simulation of *V*_signal_ + *V*_offset_ with variations in $Q_{∥}$ and $Q_{⊥}$. This result reveals that *V*_offset_ significantly contaminates the transverse output voltage under the oblique heat flux, leading to relative uncertainty in heat flux sensing. Consequently, we evaluated the impact of the difference in *S*_SE_ on the *u*_r_ of the ANT. The *u*_r_ decreases as ∆*S*_SE_/∆*S*_ANE_ decreases (Figure 4(c)), consistent with Equation (1), indicating that ∆*S*_SE_/∆*S*_ANE_ is a crucial factor in reducing *u*_r_. Furthermore, a combination of Fe_3_Ho and Fe_3_Er exhibits a ∆*S*_SE_/∆*S*_ANE_ of 0.64, whereas other combinations of reversed-sign ANE materials, (Gd_15.5_Co_84.5_/Gd_23.7_Co_76.3_, FePt/MnGa, and Fe/Fe_52_Rh_48_) [11,13,29], yields values greater than the 1.5 times values of Fe_3_Ho/Fe_3_Er (Figure 4(d)). COMSOL simulations reveal that the ANT using Fe_3_Ho and Fe_3_Er exhibits a diminutive relative uncertainty of less than 5.5% until $Q_{∥}$ reaches the same value as $Q_{⊥}$, as depicted in Figure S11(b), which is comparable to the relative uncertainty of other SE-based HFSs [30–32].

‘This study presents a sign-reversed anomalous Nernst effect with matched Seebeck coefficients in Fe_3_Ln, enabling direct and accurate heat flux sensing while minimizing offset voltage-induced relative uncertainty’..

## 4. Conclusion

This study demonstrated that compensated ferrimagnet Fe_3_Ln (Ln = Gd, Tb, Dy, Ho, and Er) offers sign-reversed *S*_ANE_ with matched *S*_SE_ for direct and accurate heat flux sensing using the ANT. The substitution with various elements (e.g. Gd, Tb, Dy, Ho, and Er) at the Ln site, while preserving the PuNi₃-type crystal structure, was confirmed by XRD measurements. Fe_3_Ho and Fe_3_Er showed *T*_comp_ at 236.3 K and 392 K, respectively, with Fe_3_Er exhibiting Fe-dominant FiM ordering at 300 K, leading to a negative *S*_ANE_, whereas the other Fe_3_Ln compounds exhibited Ln-dominant FiM ordering and a positive *S*_ANE_. The band structures and the DOS indicated that substituting Ln in Fe_3_Ln enables a sign reversal of *S*_ANE_ while maintaining *S*_SE_, ensuring minimal SE-based offset voltage and reduced *u*_r_ in heat flux sensing. Next, the sign reversal of *S*_ANE_ while maintaining *S*_SE_ was experimentally validated. The temperature dependence of *S*_ANE_ confirmed that Fe_3_Ln exhibited sign reversal above their *T*_comp_. At 300 K, Fe_3_Er exhibited a negative *S*_ANE_, while Fe_3_Ho and other Fe_3_Ln compounds maintained a positive *S*_ANE_. Despite no change in net magnetization direction, the variation in *S*_ANE_ was driven by the magnetic moment of TM atoms, as further examined by the AHE, the electrical counterpart of ANE. In contrast, *S*_SE_ remained stable across all Fe_3_Ln, with Fe_3_Ho and Fe_3_Er exhibiting only a minimal *S*_SE_ difference of 0.45 μV K^−1^ at 300 K, ensuring low SE-based offset voltage contributions. Numerical simulations revealed that the ANT composed of Fe_3_Ho and Fe_3_Er, which exhibits ∆*S*_SE_/∆*S*_ANE_ of 0.64, achieved an *u*_r_ below 5.5% until $Q_{∥}$ equals $Q_{⊥}$, comparable to SE-based HFSs. In addition to minimizing uncertainty, achieving field-free operation of ANE-based HFSs requires sufficient magnetic anisotropy to sustain remanent transverse voltage near zero magnetic field [14]. Although Fe_3_Ln compounds exhibit relatively low magnetic anisotropy compared to other TM-Ln alloys employed as ANE materials [14,21–23], this limitation may be addressed by optimizing grain structure to increase coercivity [44], thereby enabling practical field-free operation via enhanced magnetic anisotropy. These results demonstrate the potential of Fe_3_Ln-based materials for low-uncertainty ANE-based heat flux sensing.

## Supplementary Material

Supplemental Material

## Data Availability

Data supporting the findings of this study are available from the corresponding authors upon request.
