# Supplementary material for: Sign-reversed anomalous Nernst effect with matched Seebeck coefficient in lanthanide-iron alloys for the direct sensing of heat flux
Source: Sci Technol Adv Mater. 2025 Aug 7;26(1):2544649. doi: 10.1080/14686996.2025.2544649 (PMC12409908; doi:10.1080/14686996.2025.2544649)
Supplement: Supplemental Material [file TSTA_A_2544649_SM6527.docx]

**SUPPLEMENTAL MATERIAL**

Sign-reversed anomalous Nernst effect with matched Seebeck coefficient in lanthanide-iron alloys for the direct sensing of heat flux

Hyun Yu^a^, Sang J. Park^a‡^, Inho Lee^b^, Ji Hoon Shim^b^, and Hyungyu Jin^a^*

^a^Department of Mechanical Engineering, Pohang University of Science and Technology (POSTECH), Pohang 37673, South Korea

^b^Department of Chemistry, Pohang University of Science and Technology (POSTECH), Pohang 37673, South Korea

‡ Present address: National Institute for Materials Science, Tsukuba 305-0047, Japan

*Corresponding Author Contact Information:

Hyungyu Jin

Department of Mechanical Engineering, Pohang University of Science and Technology

Pohang 37673, South Korea

Tel: +82-54-279-2180 Fax: +82-54-279-5899

E-mail: [hgjin@postech.ac.kr](mailto:hgjin01@postech.ac.kr)

**Note 1 :** Derivation of the relative uncertainty


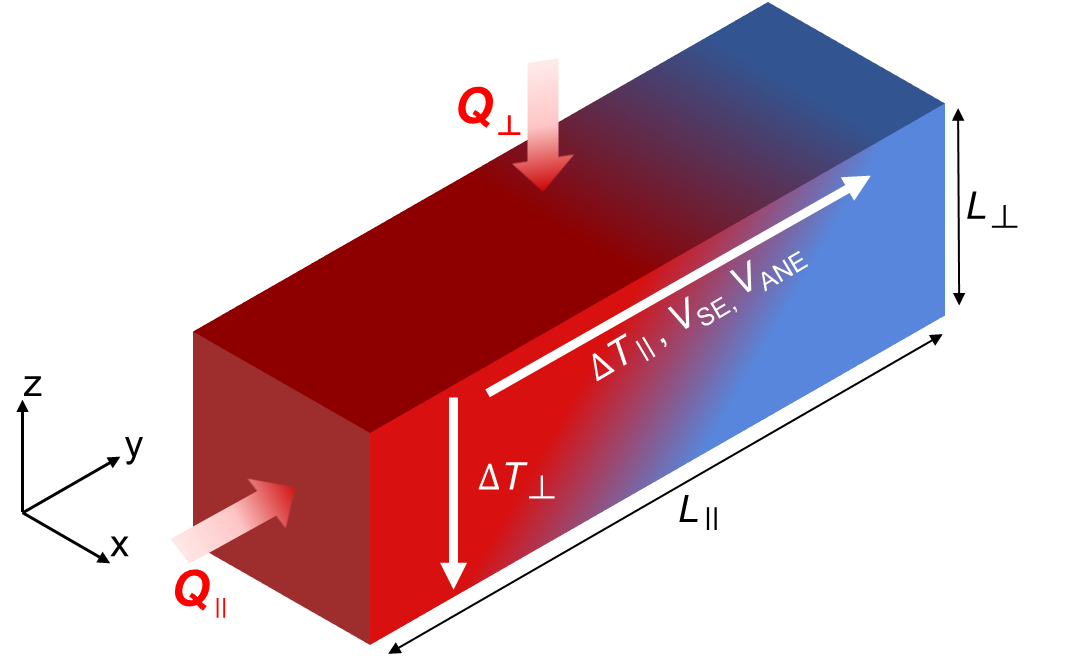


**Figure S1**. Schematic illustration of heat flow directions, temperature gradients, thermoelectric voltages, and characteristic lengths in a rectangular sample.

To derive the expression for the offset voltage-induced relative uncertainty *u*_r_, the voltage outputs generated by the Seebeck and anomalous Nernst effects are first expressed in terms of temperature gradients and characteristic lengths:

$$\frac{V_{\text{SE}}}{L_{\parallel}}=S_{\text{SE}}\times\frac{\Delta T_{\parallel}}{L_{\parallel}}$$

$\frac{V_{\text{ANE}}}{L_{\parallel}}=S_{\text{ANE}}\times\frac{\Delta T_{\perp}}{L_{\perp}}$.

Here, the subscripts $\text{∥}$ and $\text{⊥}$ denote quantities along the in-plane (e.g., y-direction) and out-of-plane (e.g., z-direction) directions, respectively, as illustrated in Figure S1. *S*_SE_ and *S*_ANE_​ represent the Seebeck and anomalous Nernst coefficients, respectively, where *L*, and Δ*T* denote characteristic length and temperature difference along the in-plane ($\text{∥}$) and out-of-plane ($\text{⊥}$) directions.

Under heat flux, differences in thermal conductivity (*κ*) between the two ANE materials constituting the ANT structure may in principle lead to unequal Δ*T* across the legs. However, in practical heat flux sensor (HFS) architectures, the Δ*T* across each leg is not determined solely by *κ* of the thermoelectric materials. This is due to the presence of heat spreaders (Al_2_O_3,_ ~24 W/mK) on both the top and bottom surfaces of the sensor, as well as highly thermally conductive metallic electrodes (Cu_,_ ~400 W/mK) that connect adjacent thermoelectric legs. These elements significantly suppress local temperature variations between legs.

This behavior was confirmed via finite element simulations performed using COMSOL Multiphysics, which showed that the local temperature differences arising from mismatch of *κ* are largely mitigated by heat spreading. The full details of this numerical analysis are provided in Note 2.

Based on this result, it is reasonable to assume that each leg experiences a nearly identical temperature difference (i.e., ${\Delta T}_{\parallel}^{1}\approx{\Delta T}_{\parallel}^{2}$​, and similarly for $\Delta T_{\perp}$​). Under this assumption, *u*_r_ can be expressed as:

$u_{r}=\frac{V_{\text{offset}}}{V_{\text{signal}}}=\frac{\Delta V_{\text{SE}}}{\Delta V_{\text{ANE}}}=\frac{\Delta S_{\text{SE}}}{\Delta S_{\text{ANE}}}\times\frac{L_{\perp}}{L_{\parallel}}\times\frac{\Delta T_{\parallel}}{\Delta T_{\perp}}$.

This expression emphasizes the importance of minimizing Δ*S*_SE_/Δ*S*_ANE_​ and optimizing device geometry (i.e., $\frac{\text{L}_{\perp}}{L_{\parallel}}$) to suppress Seebeck-induced offsets in ANE-based signal.

**Note 2 :** Numerical analysis of HFS

**
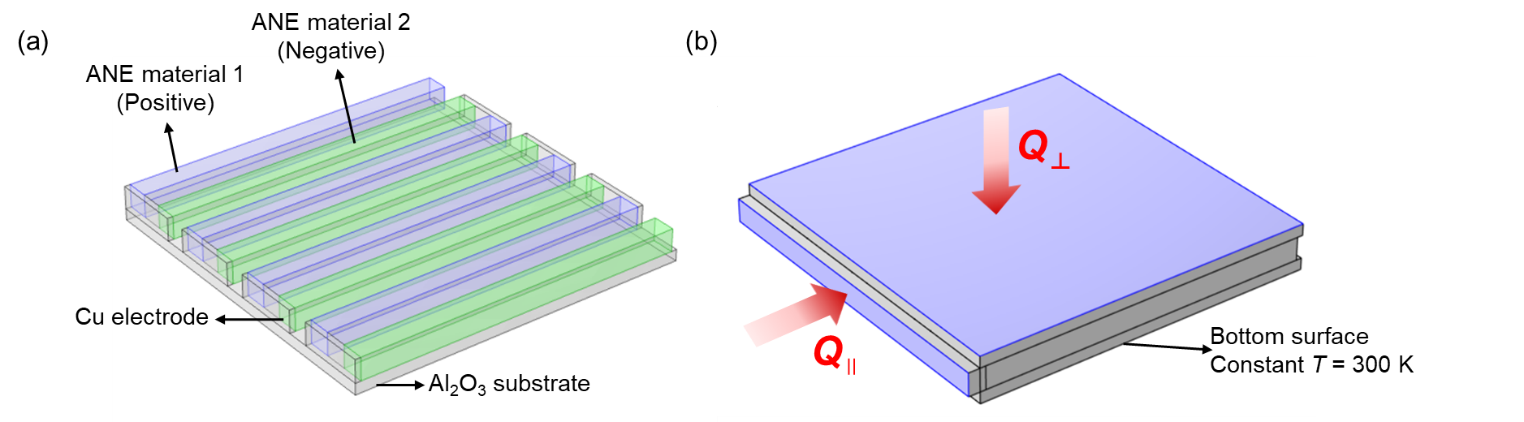
**

**Figure S2**. (a) Schematic of an ANT consisting of ANE material 1 (purple legs) and ANE material 2 (green legs). (b) Boundary conditions for numerical analysis.

Finite element simulations were performed using COMSOL Multiphysics (version 6.2) to evaluate *u*_r_​ in the ANT structure. The modeled ANT consisted of alternating positive and negative ANE materials, four legs of Fe_3_​Ho (positive ANE) and four legs of Fe_3_​Er (negative ANE), arranged as shown in Figure S2(a). Each thermoelectric (TE) leg had dimensions of 0.5 mm (width) × 14.5 mm (length) × 1 mm (height). Al_2_O_3_​ heat spreaders were attached to the top, bottom, and sides of the TE legs, and Cu electrodes were used to connect adjacent thermoelectric legs

As illustrated in Figure S2(b), perpendicular and lateral heat flows ($\text{Q}_{\perp}$​, $\text{Q}_{\parallel}$) were applied to the top and side surfaces of the heat spreader, respectively, while the bottom surface was maintained at a constant temperature of 300 K.

The thermal and electrical conductivities of Fe_3_​Ho and Fe_3_​Er were obtained from experimental data (Figure S4 and Table S1), while the material properties of Al_2​_O_3_​ and Cu were taken from the COMSOL built-in material library.

**
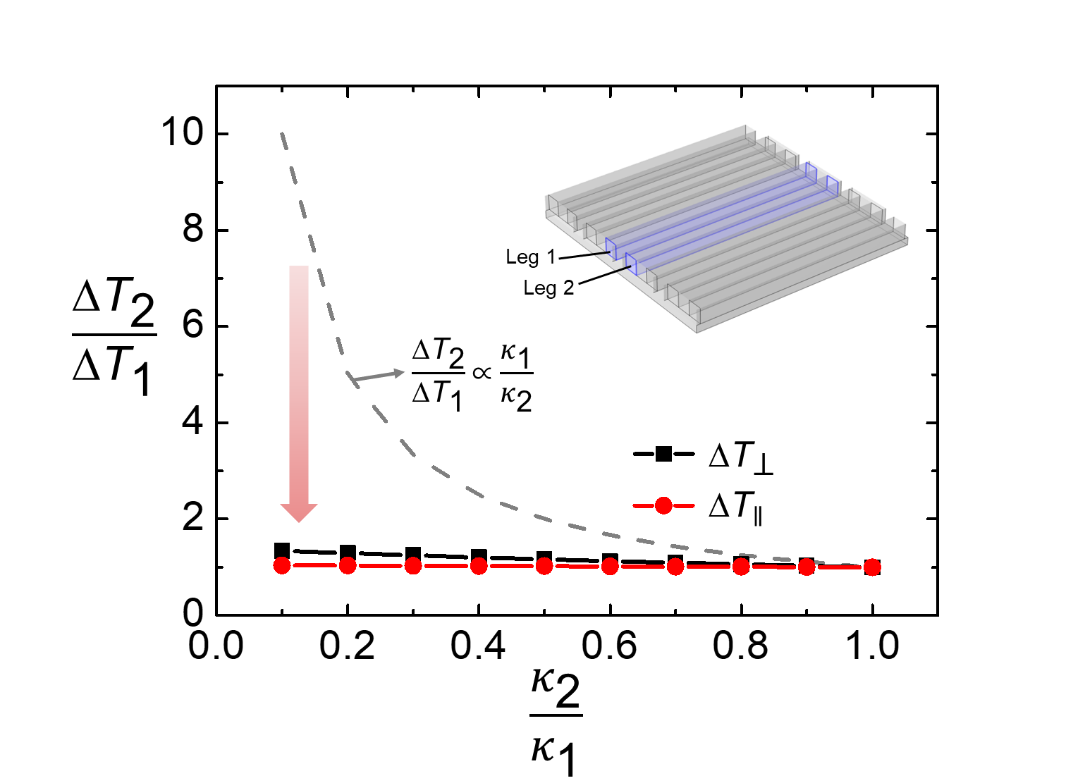
**

**Figure S3**. Simulated Δ*T*_2_/Δ*T*_1_​, as a function of *κ*_2_/*κ*_1_​ under oblique heat flux. Black circles and red squares represent the differences of out-of-plane and in-plane temperature, respectively. The gray dashed line corresponds to the ideal case without heat spreading, where Δ*T*_2_/Δ*T*_1_ $\propto$ *κ*_1_/ *κ*_2_​.

To investigate the impact of thermal conductivity mismatch on the temperature gradient between two legs in an ANT structure, we conducted finite element simulations using the geometry and boundary conditions described in Figure S2. An oblique heat flow was applied with both lateral and perpendicular components ($\text{Q}_{\parallel}$ = $\text{Q}_{\perp}$= 22.5 W), and the *κ* of Leg 1 was varied from 1 to 10 W m^-1^ K^-1^, while Leg 2 was fixed at 10 W m^-1^ K^-1^.

The Δ*T* of leg 1 and leg 2​ were extracted from the two central thermoelectric legs in the simulation domain, as indicated in the inset of Figure S3. Each value was calculated as the average temperature drop across the top and bottom surfaces (for ${\text{∆}\text{T}}_{\perp}$​) and across the left and right side surfaces (for ${\text{∆}\text{T}}_{\parallel}$​) of each leg.

Figure S3 shows the resulting Δ*T*_2_/Δ*T*_1_​ for both the out-of-plane (black squares) and in-plane (red circles) directions, plotted as a function of *κ*_2_/*κ*_1_​. The gray dashed line represents the ideal case with no heat spreading, where Δ*T*_2_/Δ*T*_1_ $\propto$ *κ*_1_/*κ*_2_, i.e, inversely proportional to *κ*_2_/*κ*_1_​. This represents the scenario in which the temperature drop across each leg is governed solely by its own *κ,* as described by Fourier’s law.

However, the simulation results clearly deviate from this idealized behavior. In both directions, the actual Δ*T*_2_/Δ*T*_1_ exhibits only a modest change, even when *κ*_1_​ is an order of magnitude smaller than *κ*_2_​. In particular, the in-plane case shows only a 3% deviation (Δ*T*_2_/Δ*T*_1_ ≈ 1.03) at *κ*_2_/*κ*_1_​=10, owing to strong thermal coupling through the electrodes. As a result The Seebeck-based offset voltage generated by the difference in ${\text{∆}\text{T}}_{\parallel}$ between the two legs is expected to be minimal, and the corresponding uncertainty associated with mismatch of *κ* is effectively suppressed.


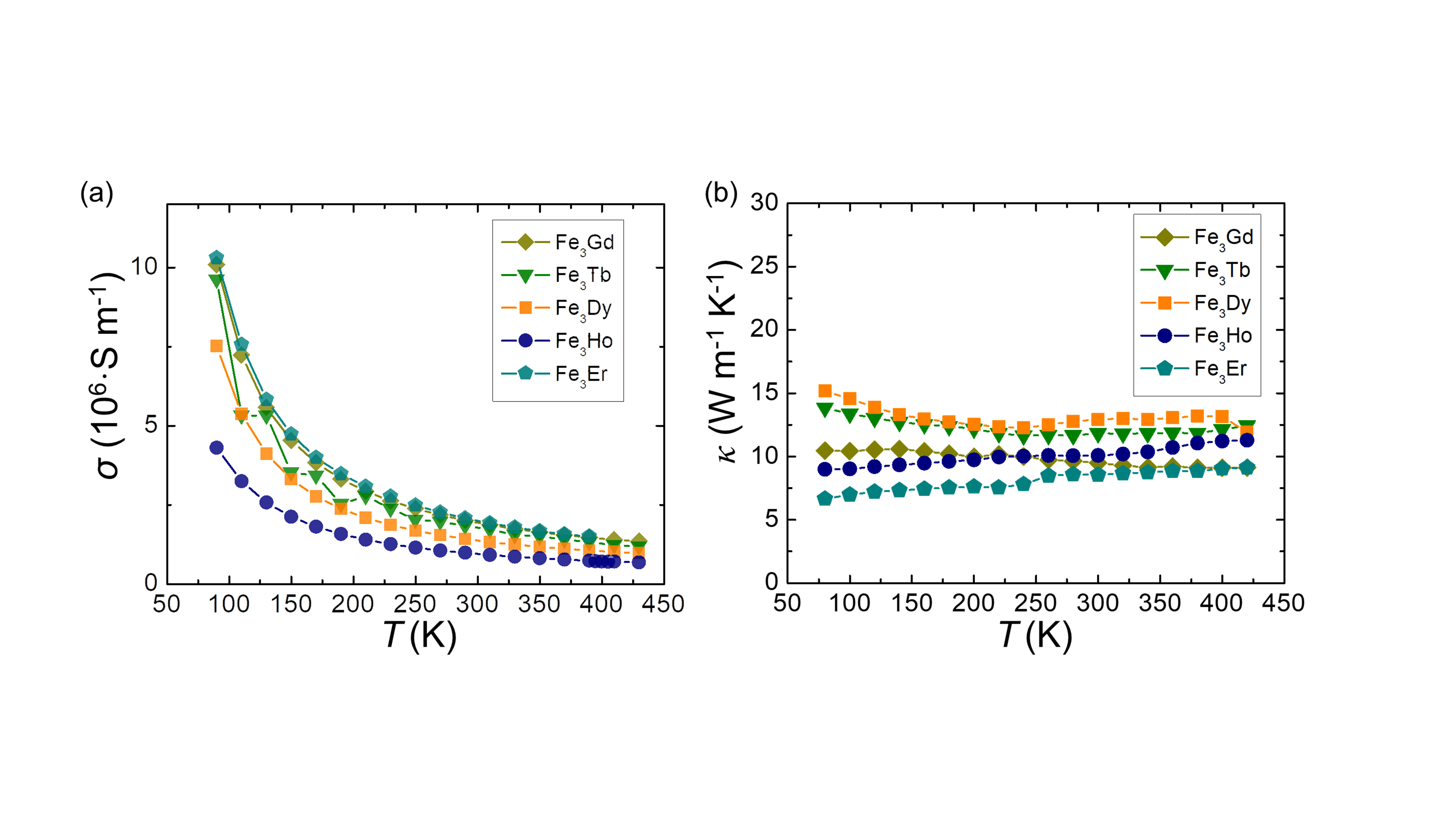


**Figure S4**. Temperature dependence of (a) electrical conductivity *σ*, and (c) thermal conductivity *κ*.

**Table S1** Material properties of Fe_3_Ln at 300 K.

|  | *S*_SE_  [μV K^-1^] | *S*_ANE_  [μV K^-1^] | *σ*  [10^6^·S m^-1^] | *κ*  [W m^-1^ K^-1^] |
| --- | --- | --- | --- | --- |
| Fe_3_Gd | -3.93 | 0.445 | 1.87 | 9.48 |
| Fe_3_Tb | -4.48 | 0.344 | 1.72 | 11.8 |
| Fe_3_Dy | -4.42 | 0.327 | 1.33 | 12.9 |
| Fe_3_Ho | -4.54 | 0.322 | 1.30 | 10.1 |
| Fe_3_Er | -4.97 | -0.288 | 1.92 | 8.54 |


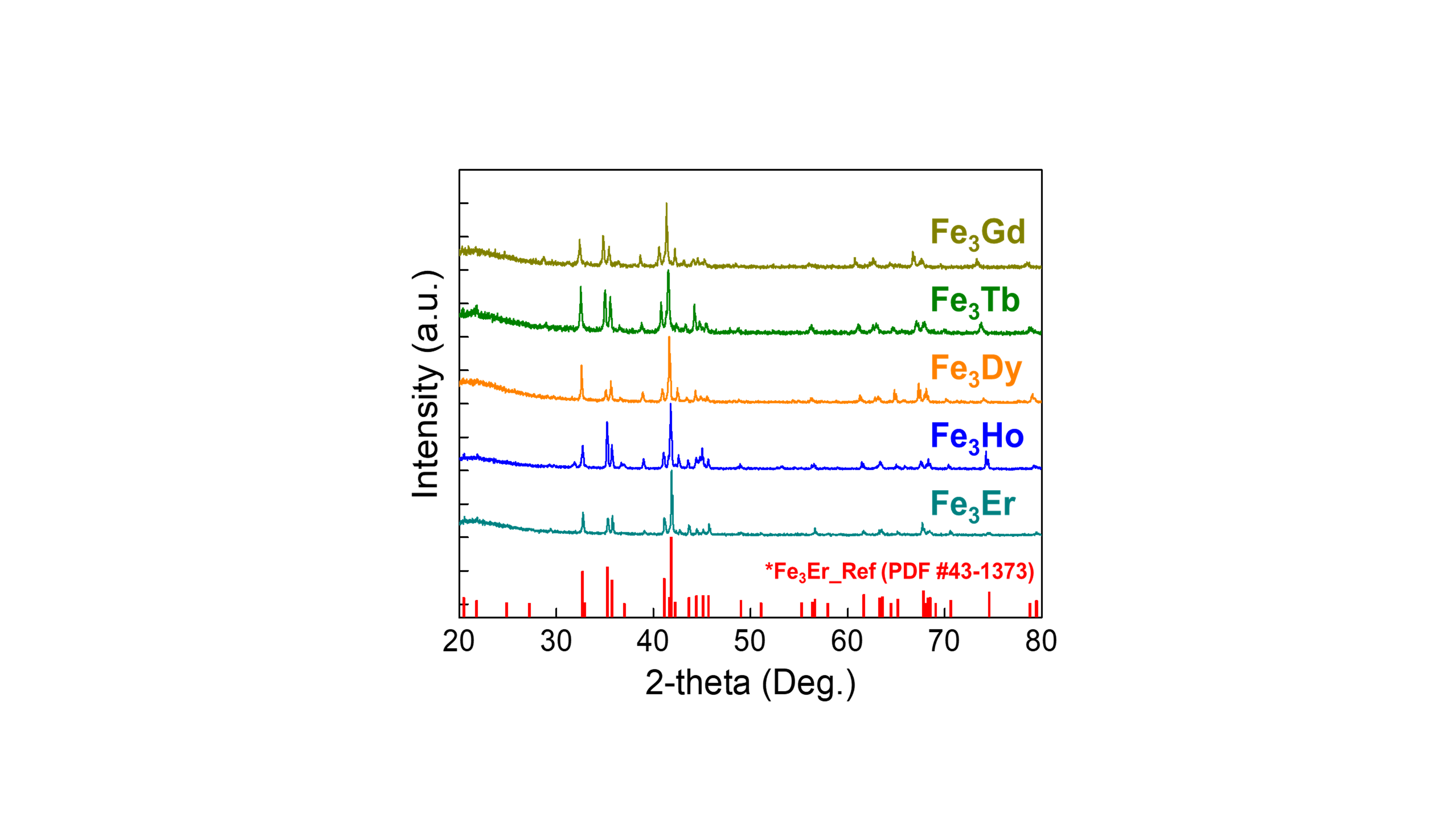


**Figure S5.** XRD patterns with reference to the XRD pattern of Fe_3_Er.


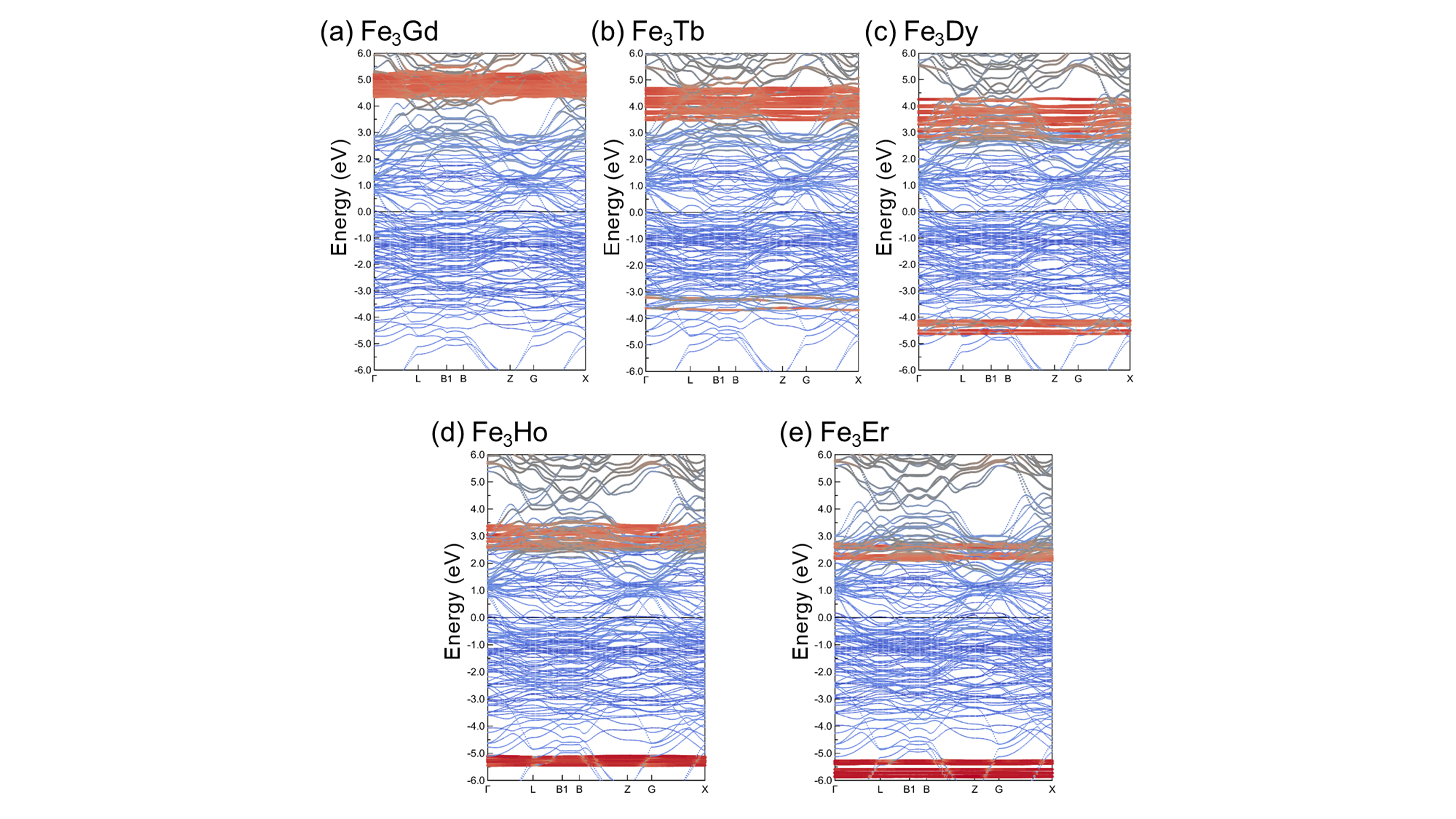


**Figure S6.** The band structures. The blue line indicates the band of Fe, and the red line indicates the band of Ln: (a) Fe_3_Gd, (b) Fe_3_Tb, (c) Fe_3_Dy, (d) Fe_3_Ho, and (e) Fe_3_Er


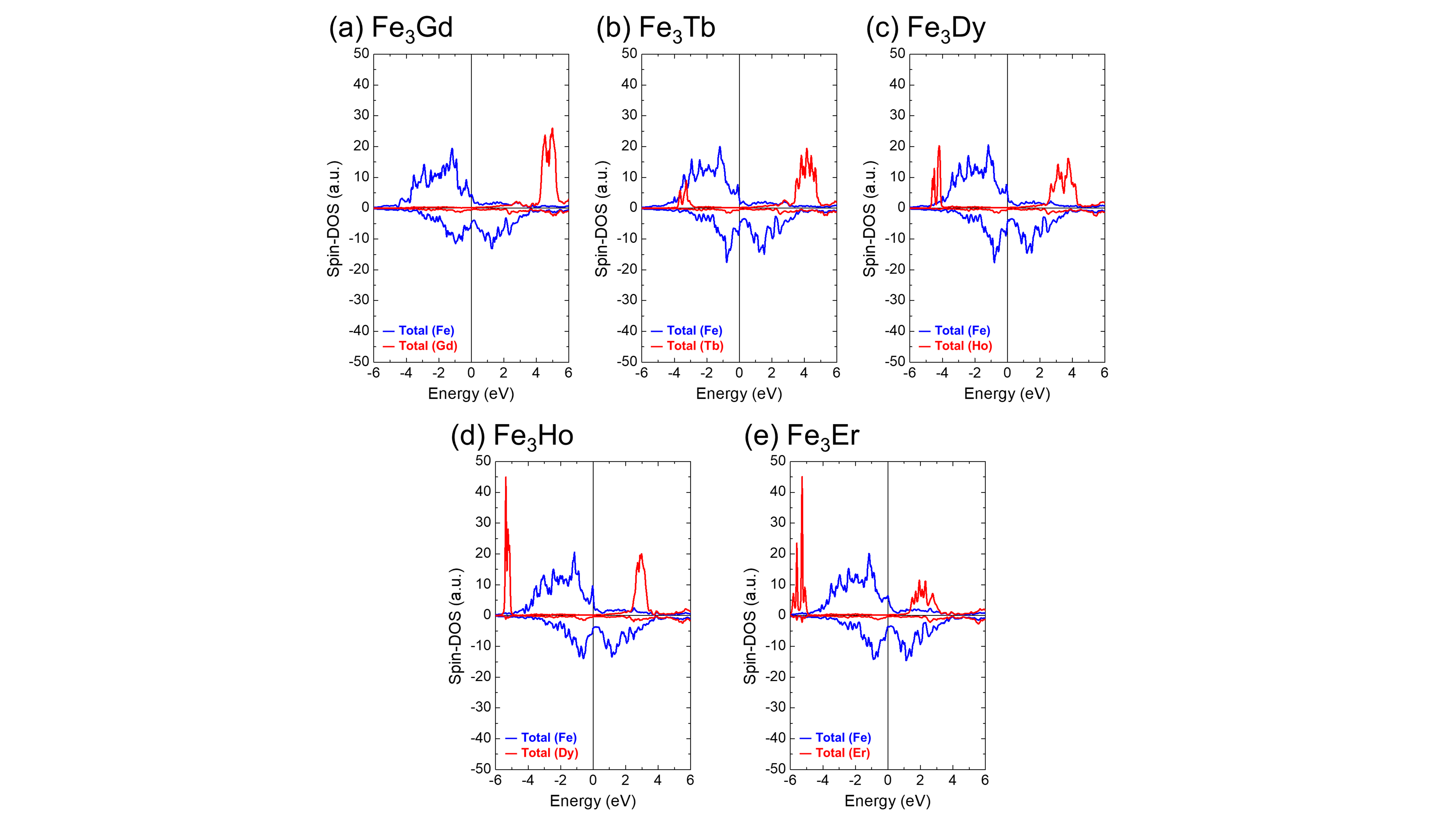


**Figure S7**. The Spin-DOS of Fe_3_Ln. Red and blue lines indicate the Spin-DOS of Fe and, respectively: (a) Fe_3_Gd, (b) Fe_3_Tb, (c) Fe_3_Dy, (d) Fe_3_Ho, and (e) Fe_3_Er.


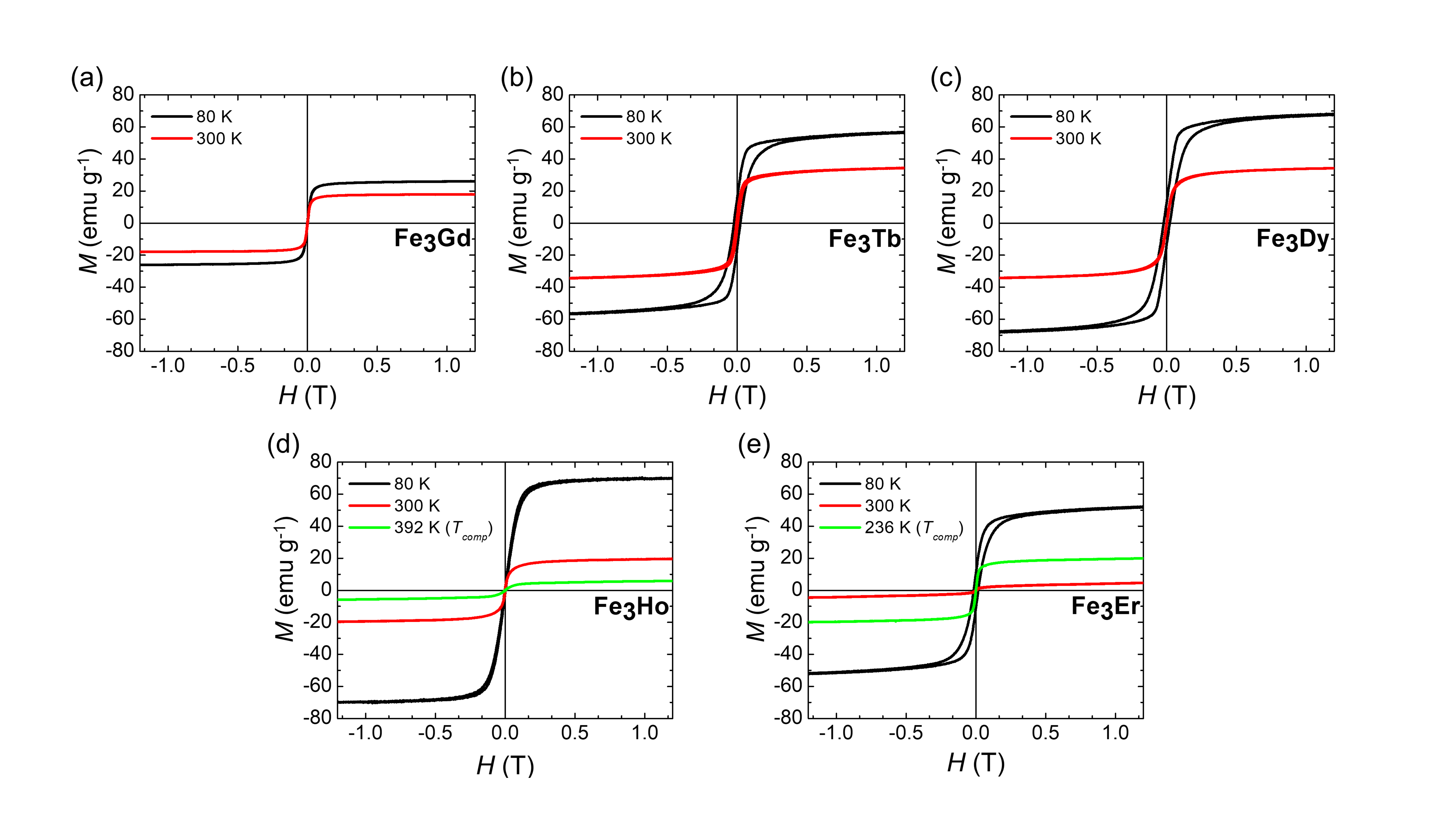


**Figure S8.** Magnetic field dependence of the magnetization at 80 K, 300 K, and *T*_comp_: (a) Fe_3_Gd, (b) Fe_3_Tb, (c) Fe_3_Dy, (d) Fe_3_Ho, and (e) Fe_3_Er.


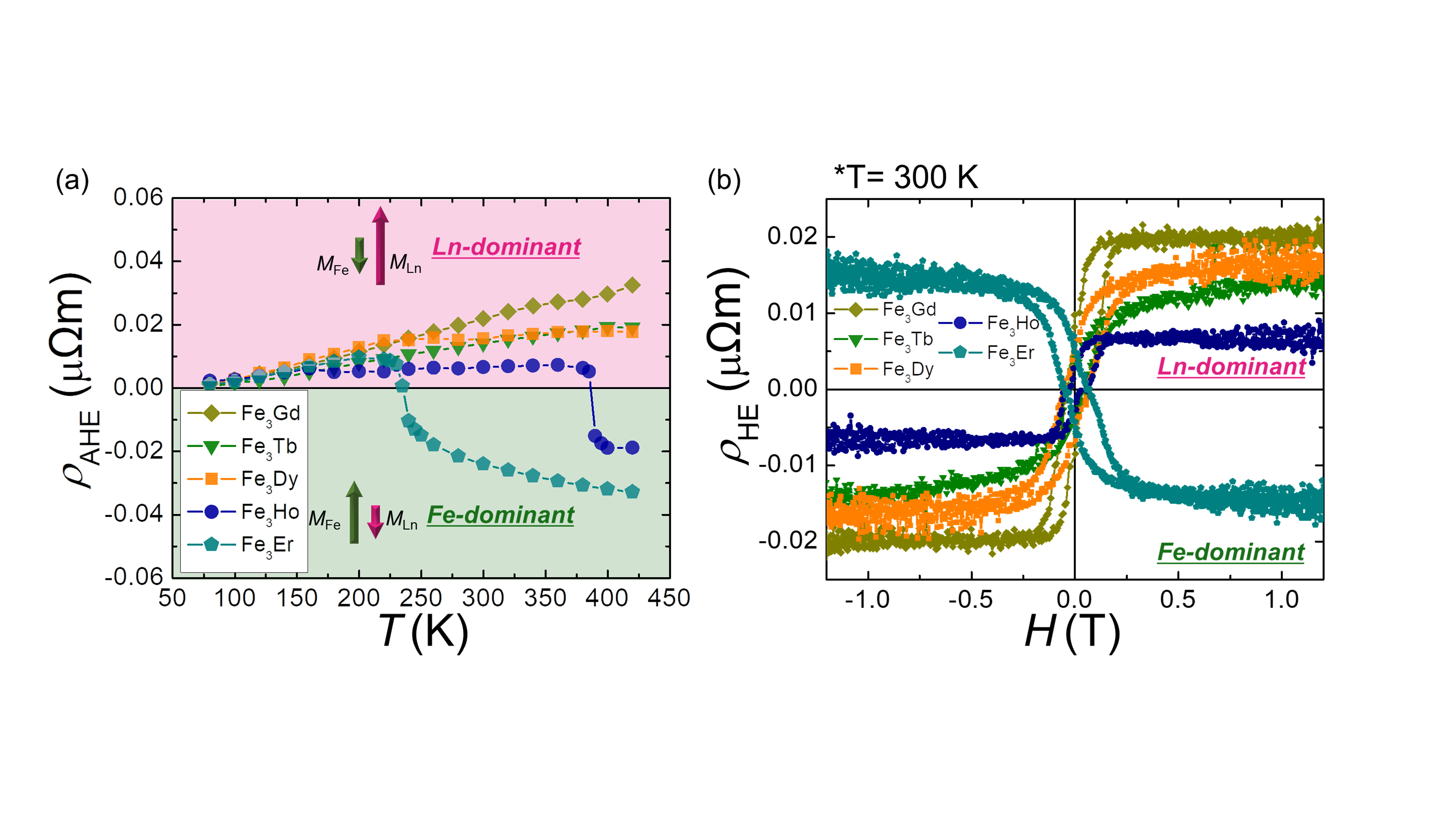


**Figure S9.** (a) Temperature dependence of the *ρ*_AHE_ and (b) magnetic field dependence of the Hall coefficient *ρ*_HE_ at 300 K.


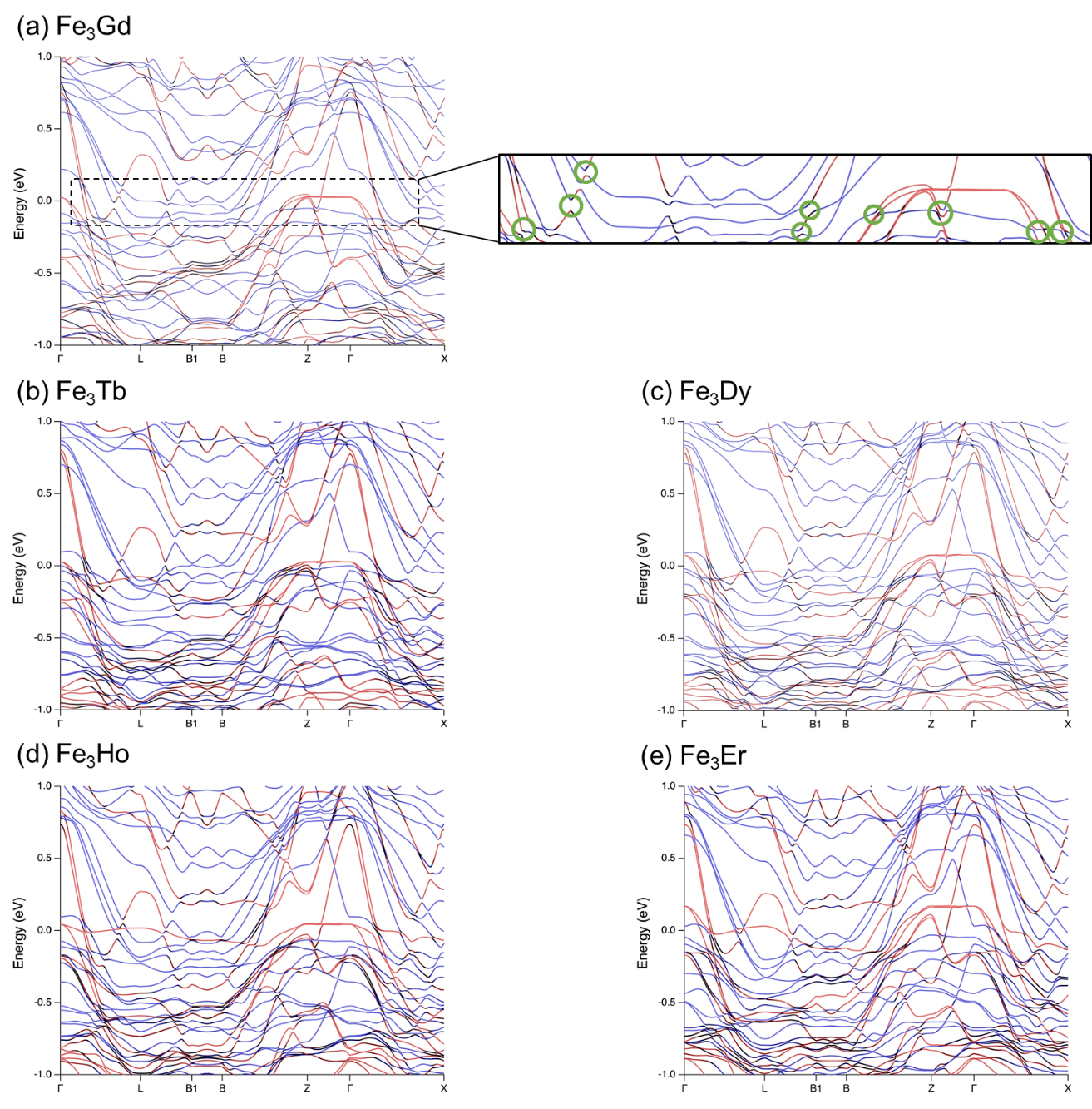


**Figure S10.** Spin-resolved band structures near the *E*_F_. Blue and red lines represent the up- and down-spin channels, respectively: (a) Fe_3_Gd, (b) Fe_3_Tb, (c) Fe_3_Dy, (d) Fe_3_Ho, and (e) Fe_3_Er. Band splittings induced by spin–orbit coupling are observed at several band crossing points, as highlighted by green circles in (a) for the Fe_3_Gd case. These SOC-induced splittings suggest the possible presence of topologically features such as Weyl points.


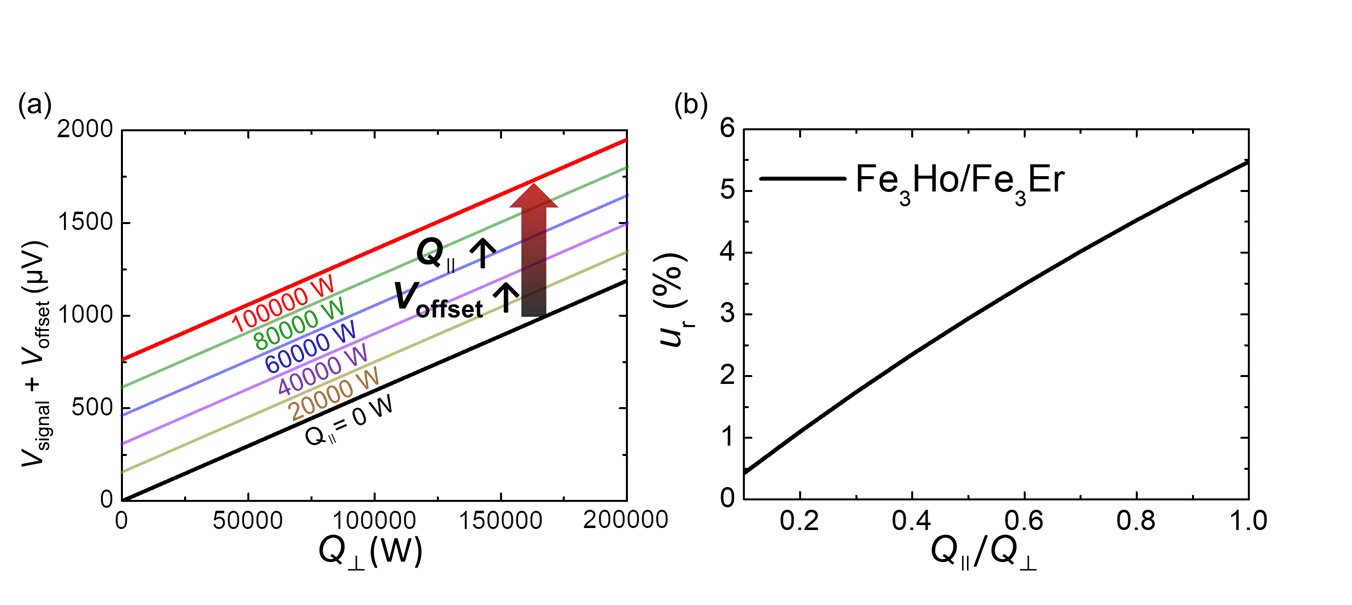


**Figure S11.** Numerical simulation of (a) *V*_signal_ + *V*_offset_ with changes in $\text{Q}_{\parallel}$ and $\text{Q}_{\perp}$ and (b) the *u*_r_ of ANT using Fe_3_Ho and Fe_3_Er with changes $\text{Q}_{\parallel}$ and $\text{Q}_{\perp}$
